# Partial Hydatidiform Mole and Coexistent Live Fetus: A Case Report and Review of the Literature

**DOI:** 10.1515/med-2019-0098

**Published:** 2019-11-10

**Authors:** Chengying Zeng, Yanbi Chen, Lijuan Zhao, Bo Wan

**Affiliations:** 1 Department of Obstetrics and Gynecology, The Third Affiliated Hospital of Southern Medical University, Guangzhou 510630, Guangdong, China

**Keywords:** Partial molar, Normal viable fetus, Prenatal diagnosis, Pseudomosaicism, Case report

## Abstract

Twin pregnancy of a hydatidiform mole with a coexistent live fetus is very rare, and complete molar pregnancy is involved in most cases. A partial molar pregnancy almost always ends in miscarriage due to a triploid fetus. Here, we report a case of a 32-year-old Chinese woman with ultrasound diagnosis of a partial molar pregnancy. Amniocentesis suggested mosaicism, but the fetus was morphologically normal. The woman chose to continue the pregnancy after fully understanding the risk. The infant was delivered prematurely, and the presence of a large single placenta with molar changes. The baby’s peripheral blood chromosomes were diploid, and the pregnant woman had no serious complications. The diagnosis, management, and monitoring of this condition will remain challenging because of its rarity. Partial hydatidiform mole combined with pregnancy can result in delivering of a normal fetus and live birth under proper management.

## 1 Introduction

Twin pregnancy with hydatidiform mole and a coexistent live fetus is a very rare condition occurring in 0.005% to 0.001% of all pregnancies [1]. Hydatidiform moles are categorized into complete and partial types that have distinct disease processes with characteristic cytogenetic, histological, and clinical features [2]. Partial hydatidiform moles (PHM) originate from dispermic fertilization of a normal haploid oocyte that generally generates a triploid set of chromosomes . A complete hydatidiform mole (CHM) contains 46 diploid paternal chromosomes. In PHM pregnancy, the fetus can develop but is malformed and non-viable, whereas no fetus develops in CHM pregnancy. Most twin pregnancies with hydatidiform mole are CHM pregnancies with a normal fetus and placenta [3]. Reports of PHM pregnancy with a live fetus are extremely rare because triploid fetuses tend to die in the first trimester [4, 5, 6, 7].

To our knowledge, only 19 cases of singleton, PHM pregnancy have been reported, five of which continued to successful delivery of a baby with diploid karyotype [8].

In this paper, we present a case of a PHM pregnancy coexisting with a diploid fetus that was later live-born. The woman presented with vaginal bleeding and a seemingly huge placenta; amniotic fluid karyotyping revealed pseudomosaicism.

## 2 Case report

On 17^th^ May 2018, a 32-year-old Hui Chinese woman, gravida 3, para 2, presented with vaginal bleeding not accompanied by uterine contractions at 22 weeks of gestation in the Department of Gynecology and Obstetrics of The Third Affiliated Hospital of Southern Medical University. The patient had no family history of gynecological or obstetric diseases and denied having taken sex hormone drugs. Ultrasonography (US) revealed a fetus with normal anatomy and an appropriate amniotic fluid volume. An abnormally thickened, single, large multicystic placenta with placenta previa was also observed. A provisional diagnosis of hydatidiform mole coexisting with a live fetus was proposed.

The serum β-human chorionic gonadotropin (β-hCG) was 169,200 mIU/mL. The patient underwent amniocentesis, and the amniotic fluid karyotype revealed was: 46, XN[51]/92, XXNN[34]. The results of quantitative fluorescence- polymerase chain reaction (QF-PCR) showed that amniotic fluid cells were chimeric with triploid and diploid, and the chimeric ratio of triploid was 10%. False mosaic phenomenon in amniocentesis had not been ruled out so we suggested performing an umbilical cord blood puncture or amniotic fluid cell fluorescence in situ hybridization (FISH) review to determine whether it was true mosaicism or pseudomosaicism, but the patient refused further examination because of economic reasons. The patient was further informed of the risk of preterm birth and trophoblastic disease, but for religious reasons, she chose to continue the pregnancy.

On 24^th^ June 2018 (29 weeks and 3 days of gestation), the patient experienced increased vaginal bleeding and abdominal pain and was admitted to hospital. Upon admission, the serum β-hCG was 153,590 mIU/mL, and the hemoglobin was 9.5 mg/dL (Mild anemia). The remaining physical examination and laboratory workup (blood pressure, serum transaminases, thyroid hormones, blood platelets and creatinine, with urinalysis, and chest X-ray examination) were normal. The baseline fetal heart rate was 140 bpm. The fetus was sensitive to non^‐^stress test. The woman received intravenous magnesium sulfate 1 g/h for 48 h and dexamethasone 6 mg q12h for two days.

**Figure 1:**
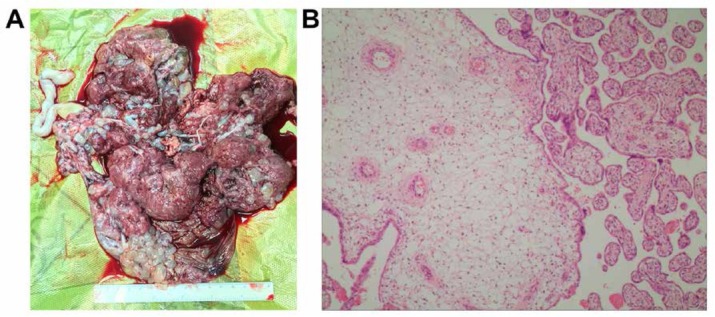
(A) The placenta was large and hydropic, with necrotic debris; (B) The trophoblastic hyperplasia of villous surfaces was limited and focally reminiscent of partial hydatidiform mole (Magnification was ×100).

Uterine contractions increased on the third day of hospitalization. The patient and her spouse were counselled on the risks and implications of the diagnosis. Under combined spinal-epidural anesthesia, a low transverse incision cesarean section was performed and a 1050 g, 38-cm long live female baby was delivered with 1, 5, and 10-minute Apgar scores of 9, 10, and 10, respectively. The placenta, which was large and hydropic, with necrotic debris, was recovered manually (Figure 1A). The estimated blood loss was approximately 400 mL. Histopathological examination of the placenta showed a mixture of large, edematous, irregular villi, combined with small normal-sized none dematous tissue. Trophoblastic hyperplasia of the villous surfaces was limited and focally reminiscent of PHM (Figure 1B).

The postpartum karyotype of the neonate revealed both 46, XX. The postoperative course was uneventful, and she recovered well and was discharged on Day 5 after the cesarean section. β-hCG concentration was 35,927 mIU/mL on the first day after the discharge; by the second week it was 2,774 mIU/mL, 420 mIU/mL by the fourth week and dropped to 0 by the tenth week. Four months after the birth, the infant weighed 4.2 kg and had a height of 53 cm. The baby is currently growing well.

**Ethical approval** The human research part of our study complied with all relevant national regulations, institutional policies, and was conducted in accordance with the tenets of the Helsinki Declaration. The present investigation was approved by the authors’ institutional review board or an equivalent committee.

**Informed consent** Informed consent was obtained from all individuals included in this study.

## 3 Discussion

Here, we report a case of a 32-year-old Chinese woman with a partial molar pregnancy and live birth of a diploid infant.

Hydatidiform mole has been known since the time of Hippocrates and is characterized by a wide spectrum of presentations and rare spectacular complications [9]. CHM consists of a diploid set of 46 chromosomes, and all genetic material is of paternal origin, with no fetal structure; it is noteworthy that the risk for trophoblastic sequelae (15%–20%) is higher than that of pregnancy with PHM (<5%) [10]. PHM pregnancies result from fertilization of an apparently normal ovum by two sperm, giving rise to a triploid karyotype (69, XXY). Three types of molar pregnancy concomitant with a normal live fetus have been identified so far, of which twin pregnancy with one normal fetus having a normal placenta and another CHM is the most common; the second type is twin pregnancy with a normal fetus and placenta and another PHM, and the third and most uncommon occurrence is a singleton normal fetus with PHM pregnancy [8].

Ultrasound is the main method for hydatidiform mole diagnosis [11]. The ultrasonographic appearance of PHM is usually honeycomb-like echo in the placenta; the boundary between the normal placental tissue and the honeycomb echo is not clear, and most fetuses are dead or malformed [12]. Few prenatal ultrasounds of PHM are structurally normal [4]. In our case, at the 22^nd^ week, the ultrasound revealed that a part of the placenta was honeycomb-like with a cyst-like echo, but the coexisting fetus showed no abnormal structures. Amniotic fluid karyotyping indicated that the fetus was a diploid/tetraploid chimera; QF-PCR confirmed diploid/triploid chimerism. Because ultrasound showed a normal fetal structure, we considered the possibility of pseudo-chimerism of the amniotic fluid. Further, the maternal and fetal conditions were monitored according to the patient’s wish. There was no significant increase in serum hCG, and the fetal growth index was normal. Finally, a live baby with normal appearance and karyotype was delivered. PHM was diagnosed by postoperative placental pathology.

The hCG level in CHM was significantly higher than that in PHM. Less than 10% of PHM patients were previously found to have hCG greater than 100 KU/L [13]. However, a high level of hCG was detected in the present case, which might have been related to the large proportion of hydatidiform mole tissue in the placenta.

Pregnancy complicated with hydatidiform mole is usually terminated immediately after diagnosis [14]. Currently, it is well understood that most CHM fetuses develop normally, and thus women can choose to continue pregnancy with appropriate support [15]. However, it is necessary to fully inform the pregnant woman of the possible maternal and fetal complications, such as preeclampsia, hyperthyroidism, vaginal bleeding, and theca lutein ovarian cysts. The probability of postpartum development into persistent trophoblastic disease is high. In cases of PHM, only few villous vesicular changes occurred; the cellular proliferation was nourished, 90% of the fetal chromosome karyotypes were triploid, and most pregnancies ended with an abortion and fetal death. The probability of postpartum development into persistent trophoblastic disease was established to be 4%, which is much lower than that for CHM; hence, chemotherapy is rarely needed, and generally no metastasis occurs [16, 17].

In the present case, the patient had slight vaginal bleeding during the second trimester of pregnancy. Her blood hCG dropped to normal one month after the delivery, and postoperative placental pathology confirmed the diagnosis. With the diagnosis of PHM we first considered that the pregnancy should be terminated. However, this case showed that PHM can result in a normal fetus and live birth under proper management. Therefore, although the incidence of this condition is very rare, this case is important because recognizing and diagnosing PHM is vital for patient care. Moreover, it should be considered and looked for in patients presenting with pre-eclampsia. Interventional prenatal diagnosis was performed by ultrasound-guided transabdominal chorionic villus biopsy and amniocentesis and combined with interphase FISH to determine fetal karyotype [18]. During this pregnancy, the condition of both mother and fetus was monitored and followed up strictly according to the principle of hydatidiform mole follow-up.

The termination of pregnancy with hydatidiform mole depends on the gestational duration and disease condition; in early pregnancy this is often associated with complete curettage of the uterine cavity. The results of the use of intra-amniotic injection of rivanol and intravenous oxytocin or cesarean section in the second trimester of pregnancy are controversial [19]. Caesarean section is recommended to deliver a live fetus with hydatidiform mole, as repeated uterine contraction increases the possibility of squeezing of the hydatidiform tissue into the abdominal cavity, leading to increased risk of pulmonary embolism. Due to placenta previa and premature labor, the patient enrolled in this study chose lower uterine segment cesarean section. Oxytocin was used after the rapid removal of the grape tissue during the operation. The uterine contraction was good, with little bleeding.

## 4 Conclusion

In this report, we have described a rare case of partial hydatidiform molar pregnancy coexistent with a live fetus with pseudomosaicism. This case shows that under proper management, PHM combined with a live fetus can result in live delivery of a normal infant.
